# Making every contact count with seldom‐heard groups? A qualitative evaluation of voluntary and community sector (VCS) implementation of a public health behaviour change programme in England

**DOI:** 10.1111/hsc.13764

**Published:** 2022-02-26

**Authors:** Deborah Harrison, Rob Wilson, Andy Graham, Kristina Brown, Hannah Hesselgreaves, Malgorzata Ciesielska

**Affiliations:** ^1^ Newcastle Business School Northumbria University Newcastle‐upon‐Tyne UK; ^2^ Public Health Consultant Local Government North East England UK

**Keywords:** behaviour change, health policy, health promotion, health services research, public health, qualitative research, voluntary and community sector

## Abstract

*Making Every Contact Count* (MECC) is a national, long‐term public health strategy in England. It supports public‐facing workers to use opportunities during routine contacts to enable healthy lifestyle changes. This paper reports the findings from an external evaluation of voluntary and community sector (VCS) delivery of MECC in the North East of England, which focused on engaging under‐represented client groups. The study aimed to (a) Establish if (and how) MECC had impacted the workforce, including changes to staff knowledge, confidence and behaviour; (b) Identify benefits, challenges and unintended consequences; and (c) Explore outcomes for service users. A multi‐stage qualitative design focused on understanding both process and outcomes. The study utilised three data collection methods, including a journey mapping workshop (*n* = 20), semi‐structured interviews with delivery leads, VCS workers and volunteers who had accessed MECC training (*n* = 11), and focus group discussions with clients (*n* = 22). The findings illustrated positive early outcomes, including improvements in self‐reported staff knowledge and confidence as well as emerging examples of organisational culture shift and individual behaviour change. Alongside this, the data provided a rich picture of barriers and challenges which are examined at different levels—national programme, local programme, VCS sector, partner organisation, worker and client. The research highlights clear successes of the VCS delivery model. However, it is presented as a ‘double‐edged sword,’ in light of associated challenges such as sector‐level funding uncertainty and accessibility of MECC resources to diverse client groups. The discussion considers issues related to the measurement and attribution of behaviour change outcomes for brief interventions, as well as fidelity, legacy and long‐term sustainability challenges. The recommendations call for system‐level analysis and comparison of different MECC implementation models, to improve our understanding of challenges, opportunities and programme reach for behaviour change intervention programmes—particularly in relation to seldom‐heard client groups.

**What is known about this topic**

- Interventions focused on health behaviours such as diet, physical activity, smoking and alcohol are considered central to improving health.
- MECC supports health and social care practitioners to deliver brief interventions, through training and resources.
- There is limited understanding of outcomes and challenges for practitioners, organisations and clients—particularly in non‐clinical contexts and with under‐represented client groups.

**What this paper adds**

- MECC led to positive outcomes including improved staff knowledge and confidence, organisational culture shift and individual behaviour change.
- Advantages of VCS delivery included the breadth of client groups accessing interventions, while challenges included funding uncertainty and accessibility of MECC resources.
- Wider discussion includes long‐term sustainability challenges, support requirements and barriers to measuring outcomes.

## 1 INTRODUCTION

### 1.1 Policy context

Individual and collective behaviour change has emerged as a key priority in tackling chronic illness and improving population health across Europe (WHO, 2012). In 2014 it was estimated that health factors such as smoking, alcohol, physical inactivity and being overweight cost the NHS over £12.6 billion per year (NICE, 2014). Detrimental health behaviours also impact local economies through reduced productivity, sickness absence and increased demand for social care (Beard et al., 2019).

Research shows that opportunistic behaviour change interventions delivered by health and social care practitioners during routine contacts with clients are cost‐effective and can reduce local health inequalities (PHE, 2019a,b). Such interventions are becoming part of the remit of an increasing spectrum of workers across health, social care and wider public services (Byrne‐Davis et al., 2018).

*Making Every Contact Count* (MECC) is a long‐term public health strategy, rolled out in NHS Trusts and local authorities across England since 2010. It supports public‐facing workers to, ‘use opportunities during routine contacts to support, encourage and enable people to consider healthy behaviour changes’ (PHE, 2016:5). MECC targets a range of health factors including smoking, diet, physical activity, alcohol use, mental health and wellbeing (HEE, 2021). It involves frontline workers initiating brief, health‐related conversations as part of routine appointments and—where appropriate—signposting to local services and information:

‘The ultimate aim is to make health‐related behaviour change interventions commonplace in a wide range of settings within and beyond the NHS.’ (Nelson et al., 2012:655)

A key focus of MECC is on developing staff competencies and organisational processes, through the provision of training and materials, to enable effective interventions to take place (HEE, 2021).

### 1.2 Existing literature

Despite its prominence as a national public health strategy, there has been limited academic research published on the implementation and impact of MECC. Evidence is predominantly drawn from small‐scale studies in clinical or other healthcare contexts, with a central focus on process evaluation rather than impact. Difficulties assessing outcomes have been highlighted as particularly problematic, with researchers often relying on indirect staff feedback or compiled case studies to draw conclusions about client outcomes (Nelson et al., 2012; Patten & Crutchfield, 2016). Evaluation of MECC in local government contexts has focused on internal staff groups such as housing officers or social workers, rather than wider community organisations (Dewhirst & Speller, 2015).

Despite its limited scope, the existing literature illustrates positive early experiences of MECC implementation. Nelson et al's. (2012) interview‐based study of NHS and public health practitioners in two English regions identified strengths including MECC’s simplicity, flexibility and low cost. Reported organisational benefits include a positive impact on perceived organisational culture and increased team‐bonding opportunities (Dewhirst & Speller, 2015), alongside changes to operational systems and practice (Patten & Crutchfield, 2016). For frontline workers, findings demonstrate improved skills, knowledge and confidence measured using pre‐ and post‐training evaluation questionnaires—for both MECC and similar behaviour change programmes (Bull & Dale, 2020; Dewhirst & Speller, 2015; Patten & Crutchfield, 2016). However, the sustainability of specific techniques included in MECC training, such as open discovery questions and goal‐setting, seems to be more varied (Frost et al., 2018; Lawrence et al., 2016).

Existing studies illustrate variable uptake of MECC, which is explained by a range of practical, attitudinal and cultural barriers (Keyworth et al., 2018). Examples include resistance from medical practitioners, unease about the potential to offend clients and staff concerns over workload increases related to recording, referral and monitoring requirements (Keyworth et al., 2018; Nelson et al., 2012; Patten & Crutchfield, 2016). Dewhirst and Speller (2015) highlighted that, while staff knowledge and confidence increased as a result of MECC, there was little change to the wider factors that make discussing healthy lifestyles easier or more difficult—such as time available, client attitudes and service organisation. Greater exploration of factors affecting the use of behaviour change interventions post‐training, including workplace barriers and the availability of support, is needed (Bull & Dale, 2020).

### 1.3 The role of the VCS in health and social care delivery

The last few decades have seen a significant increase in the delivery of public services by the voluntary and community sector (VCS) in the UK and internationally (Dacombe & Morrow, 2016; Newbigging et al., 2017). Research shows that VCS agencies are well‐placed to cross institutional boundaries in health and social care and increase capacity related to health promotion, yet they experience challenges related to training needs, resources and lack of systematic approaches to outcomes evaluation (Boyle et al., 2007; Croft & Currie, 2020). Findings also highlight the challenges of partnership working between VCS and statutory functions such as general practice, including differences in operational systems, governance and professional boundaries (Southby & Gamsu, 2017).

A unique and distinctive feature of the VCS role in public service delivery has been the ability to gain the trust of under‐represented groups, enabling engagement with—and playing an important role in the provision of services for—those considered ‘seldom‐heard’, ‘marginalised’ or ‘hard‐to‐reach’ (Flanagan & Hancock, 2010; Healthwatch, 2020; Powell et al., 2017). Such phrases are used to describe groups who have traditionally been excluded from, or inadequately represented in, services or decision‐making. Examples include ethnic minority groups, carers, the LGBTQ + community, people with mental or physical disabilities, refugees and asylum seekers, people experiencing homelessness and young people (Flanagan & Hancock, 2010; Healthwatch, 2020).

Barriers to accessing services for this cohort are well‐documented. Findings highlight a need for flexible service boundaries, increased partnership working and time to establish trust in order to improve service experience and engagement (Flanagan & Hancock, 2010). It is argued that the greater flexibility and ability to establish trust by the VCS has led to its relative success in engaging under‐represented groups, when compared to more mainstream approaches (Flanagan & Hancock, 2010; Goopy & Kassan, 2019; Powell et al., 2017).

The challenges of realising the policy goal of greater VCS involvement in health and social care delivery has led to initiatives such as financial incentives and the creation of new workforce roles and network structures to support VCS engagement (Croft & Currie, 2020; Isaacs & Jellink, 2007; Jennings, 2015). A recent study of voluntary sector involvement in integrated care provision by Croft and Currie (2020) illustrated the value of dedicated workforce roles in facilitating joint working and supporting VCS agencies to navigate the complex system barriers between health and social care. The findings highlighted the importance of regulation and ‘normative control’ exerted by commissioners, to maximise engagement from healthcare professionals and reduce the risk of exploitation of VSC provider flexibility to ‘patch’ provision by overstretched service providers.

### 1.4 The current study

This paper broadens the evidence base by drawing upon findings from an external evaluation of voluntary and community sector (VCS) implementation of MECC. The evaluation took place from February to July 2019. Given the policy priorities already described, this context provided a unique setting within which to explore MECC implementation and early impact on stakeholders including partner organisations, frontline workers and clients.

The aims of the research were to:

- Establish if (and how) the programme had impacted upon the VCS workforce.
- Identify any benefits, challenges and unintended consequences.
- Explore the extent to which MECC had influenced outcomes for end users.

### 1.5 Research context

The evaluated programme was funded from June 2017 to September 2019 by a local authority in the North East of England. It involved the delivery of training in MECC brief interventions to over 500 frontline staff, alongside a £300,000 grant fund made available to local VCS organisations to enable participation and embedding of the MECC approach. The programme design targeted VCS organisations who were working with seldom‐heard groups such as asylum seekers, carers and people with learning difficulties, as well as those at heightened risk of wider health inequalities such as cancer survivors. A total of 19 local VCS partners were involved initially, forming the primary focus for the research. Later phases extended to wider council services and additional groups including the LGBTQ + community and armed forces service leavers.

## 2 METHODS

### 2.1 Study design

The research utilised a three‐stage, qualitative design:

1. Journey mapping workshop with the MECC delivery team, VCS leads, workers and volunteers (*n* = 20).
2. Semi‐structured interviews with MECC delivery team members, VCS leads, frontline workers and volunteers (*n* = 11).
3. Focus group discussions with service users (*n* = 22).

The combination of methods allowed a range of perspectives to be sought and was considered important to maximise validity of the findings through triangulation (Patton, 2002). The study received ethical approval from Northumbria University (ref 15553). Written informed consent was obtained from participants at the start of the workshop, interviews and focus groups. Discussion prompts for each stage are provided in Table 1.

**TABLE 1:** Overview of discussion topics for the three data collection phases

Stage 1 Mapping workshop	Mapping prompts *WHERE YOU STARTED* (Expectations—Resources—People involved—Plans for funding—Motivation to be involved—What you wanted to achieve) *WHERE YOU ARE NOW* (Activities—Resources—Staff groups/partners involved—How funding is used—Motivation—What you have achieved) *THE PROCESS* (Decisions made—Changes of plans—Challenges—Operating context—Expectations versus reality) What have you learned? Where next? Table discussion prompts 1. What are the main differences between ‘where you started’ and ‘where you are now’? Reasons for those differences? 2. Anything you would do differently now? 3. How similar or different are the experiences around your table?
Stage 2 Interviews	1. Your role and involvement with MECC 2. Early days and expectations *[How did you feel about getting involved? What barriers/facilitators did you face during initial implementation?]* 3. MECC in your organisation *[What does MECC look like in your organisation now? How have staff responded? Any changes to organisational processes? How is information about MECC recorded?]* 4. Impact or benefits of MECC *[For staff, organisation, clients, wider organisations/networks]* 5. Challenges and barriers *[For staff, organisation, clients]* 6. Any ways MECC could be modified or improved? 7. Do you plan to continue MECC within the organisation?
Stage 3 Client focus groups	1. What do you think about the health and wellbeing (MECC) activities/sessions you have taken part in recently? *What did you like about them? Anything you didn't like? Anything that could be improved?* 2. Have any changes happened as a result of the sessions? *Are you doing anything differently? Any new skills or things you've learned? Any other changes that have happened since?* 3. Do you have any recommendations that you would like to make? *What could improve the sessions/activities? Anything else you would like to see in your local area related to health and wellbeing?*

### 2.2 Sampling

A purposive sampling approach was taken whereby prospective participants were approached due to their involvement in MECC as workers, volunteers or clients. All 19 VCS organisations were approached to take part via an opt‐in invitation email, sent out by the MECC programme on behalf of the research team in order to uphold data protection requirements. Separate invitation emails were sent regarding each study element (including the mapping workshop, interviews, focus groups and a final dissemination event), approximately 6–8 weeks apart.

Table 2 provides an overview of the organisational focus and core client group of participating VCS organisations, alongside an indication of their level of involvement in the study. 13 VCS partners took part in at least one element of primary data collection, alongside one internal council department. The focus of organisations for those who did not take part included homelessness, dementia, eating disorders, poverty and learning difficulties. Where reasons for non‐participation were provided these included staffing and capacity challenges, particularly for smaller organisations. Table 3 provides an overview of key sample details and data generated at each research stage.

**TABLE 2:** Overview of organisational focus, client group and level of involvement in the study for participating VCS organisations

Identifier	Organisation focus/client group	Participation in study
VCS01	Young adults Learning disabilities/difficulties Autism spectrum disorder (ASD) Mental health	MW, I, FG (*n* = 9)
VCS02	Carers Young carers	MW
VCS03	Cancer support	MW, I
VCS04	LGBTQ+ Social inclusion and mental health	MW
VCS05	Disadvantaged children and young people Social inclusion	MW
VCS06	Community cohesion and antisocial behaviour Young people	MW
VCS07	Learning disabilities/difficulties Autism spectrum disorder (ASD) Mental health Advocacy and user voice	MW, I, FG (*n *= 7)
VCS08	Consumer rights Financial, housing, employment, health, immigration, family law	MW, I
VCS09	Minority ethnic groups Arts and cultural development	MW
VCS10	Mental health Older people Children and young people	MW, I, FG (*n* = 6)
VCS11	Refugees and asylum seekers	MW
VCS12	VCS infrastructure organisation Sector advice and support	I
VCS13	Young women and young mothers Crisis support	I

Note

Key for study involvement indicators in Table 2: Mapping workshop [MW], VCS interviews [I] and client focus group [FG].

**TABLE 3:** Sample details and data generated for the three data collection phases

Study element	Number of participants	Participant details	Data generated
Stage 1: Mapping workshop	20 (15 from 11 VCS partners; 5 local authority MECC delivery leads)	7 female; 13 male Job roles including Chief Executive, Development Manager, Advocacy Worker, Volunteer Coordinator, member *See Table 2 for client groups represented*	Written VCS and MECC team ‘journey maps’ Detailed table discussion notes
Stage 2: Interviews	11 (6 VCS delivery leads and workers; 3 MECC local authority delivery leads; 2 from an internal council department participating in MECC)	6 female; 3 male Job roles including Chief Executive, Partnership Support Manager, Outreach Worker and User Involvement Worker *See Table 2 for client groups represented*	Interview transcripts Field notes
Stage 3: Client focus groups	22 (From 3 VCS partner organisations)	11 female; 11 male Age range from 18 to over 80 years Focus Group 1 (*n* = 9)—Young people with learning disabilities and difficulties Focus Group 2 (*n* = 7)—Adults with learning difficulties, autism spectrum disorder (ASD) and mental health support needs Focus Group 3 (*n* = 6)—Older people's mental health group	Focus group transcripts (2) Detailed discussion notes (1) Field notes

### 2.3 Stage 1: Mapping workshop with MECC delivery team, VCS leads and frontline workers

An initial, face‐to‐face journey mapping workshop brought together VCS service managers, frontline workers and volunteers (*n* = 15 from 11 VCS organisations) with the local authority MECC delivery team (*n* = 5). Participants undertook a ‘MECC Journey Mapping’ exercise to prompt reflection on the implementation process, including decisions made and challenges faced. 16 individual maps were generated—one for each participating VCS organisation and one for each MECC delivery team member. Semi‐structured table discussions facilitated by the research team further explored the experiences of attendees, documented through detailed written notes.

### 2.4 Stage 2: Semi‐structured interviews with delivery team, VCS leads and frontline workers

Stage 2 explored the views of delivery leads, frontline workers and volunteers in more depth. One‐to‐one semi‐structured interviews (*n* = 11) were undertaken with local authority MECC delivery leads (*n* = 3), VCS MECC leads and frontline workers (*n* = 6) and one internal council department (*n =* 2). The majority of interviews took place in a private meeting room at the participant's place of work, with one conducted via telephone and one on university premises according to participant preferences. Interviews were audio‐recorded and lasted from 34 to 95 min.

### 2.5 Stage 3: Focus groups with clients and service users

Three focus groups took place during June‐July 2019, involving 22 clients plus 3 supporting frontline workers. All 19 organisations were invited to take part, of which 3 opted in during the study timeframe. Each focus group was facilitated by two researchers and took place on the VCS organisation's premises, as part of a regular group meeting. Study information was sent ahead to group facilitators, alongside an offer to discuss any specific requirements or attend an earlier meeting to introduce the research team in advance. Materials were adapted where required, for example through the development of large‐print study information sheets and shortened discussion questions. Focus groups lasted between 49 and 79 min, with two of the three being audio‐recorded. In the remaining group, detailed notes were taken.

Focus group participants represented a range of seldom‐heard groups including those with learning disabilities and difficulties, young people, older people and mental health service users. Participant status as a member of an under‐represented group was inferred from the person's group membership (for example being a member of a learning difficulties group), rather than the collection of detailed demographic or needs‐related information from individual participants. Related ethical considerations and limitations are considered in the discussion section.

### 2.6 Analysis

The data was analysed thematically using an approach based on Ritchie and Spencer’s (1994) thematic framework analysis. This involves a series of processes including familiarisation, indexing, framework development, mapping and interpretation. Facilitated using the QSR analysis software NVivo, an ‘open coding’ process was used to iteratively generate a thematic framework from the data. Mapping, interview and focus group data were initially coded separately; however, emerging themes were later merged into one shared framework due to a high level of correspondence between themes identified across the different data sources. An overview of themes and sub‐themes generated through the analysis is provided in Table 4.

**TABLE 4:** Thematic framework (including themes and sub‐themes)

Theme	Sub‐theme	Example codes
1. Early days and expectations	1.1. *Motivation and partner expectations*	Compatibility with goals/ethos/areas of interest (high) Maximise value of everyday contacts Partnership/consortium/‘joined‐up’ approach Training opportunities Mixed expectations
	1.2. *Clarity (lack of)*	Ambiguity Uncertainty/confusion
2. Organisational approach and delivery model	2.1. *Programme reach and diversity*	Whole workforce approach Partners involved (range of) Areas of focus (range of) Groups reached (range of) Close relationships (to client)
	2.2. *Variations in implementation*	Flexibility Structure of MECC Activities delivered (range of)
	2.3. *MECC programme team and resources*	Team (positive) Training (mixed) Tailoring resources (need for/process of) Planning and administration
	2.4. *Partnerships and collaborative working*	Networking (value of) Formal opportunities (fewer than anticipated)
3. Organisational outcomes and impact	3.1. *Reinforcing existing practice*	‘Already doing it’ Framework/scaffolding Consistency of approach (improved) ‘Validation’ of practice Visibility to funders (improved)
	3.2. *Impact on organisations*	Processes Culture Cumulative impact of strengthened staff practice ‘Catalyst’
	3.3. *Wider outcomes*	Building networks Accessing funding
4. Worker outcomes	4.1. *Knowledge, confidence and motivation*	Improvements in Lack of change/Individual differences
	4.2. *Changes to practice*	Client interactions and signposting Being proactive Attitude change
	4.3. *Impact on health and wellbeing (self or others)*	Lifestyle changes ‘Mindful’ choices Self‐care (mental health) Setting example to friends/family Attitude/response to family/friends (more positive)
5. Client outcomes	5.1. *Knowledge and awareness*	Healthy habits ‘Shock factor’ Food and nutrition Physical activity Other (alcohol, mental health, sleep, daily routines, budgeting)
	5.2. *Behaviour change*	Food and nutrition Physical activity Other behaviour change
	5.3. *Wider outcomes*	Social networks and community inclusion Peer facilitation and communication skills Wider community impact
6. Barriers, challenges and individual differences	6.1. *Programme and partner‐level*	Accessibility/appropriateness of MECC resources Organisational commitment (variations in) Local constraints VCS challenges Legacy and long‐term knowledge transfer Measuring outcomes
	6.2. *Worker‐level*	Individual differences Background Role and service focus Time/workload pressures Perceptions of worker‐client relationship
	6.3. *Client‐level*	Individual differences Complexity Wider circumstances Long‐term support (importance of)

Emerging findings and research team interpretations were shared with the local authority programme team (*n* = 5) for discussion and interrogation at a face‐to‐face session in June 2019. Following this they were shared with VCS partners, clients and wider stakeholders for feedback and discussion, as part of a MECC celebration event in autumn 2019 (*n* = 69).

## 3 Findings

The findings reported here focus on VCS implementation and delivery of MECC. We examine key features and advantages of VCS delivery, before exploring outcomes for organisations, workers and clients. The final section outlines barriers and challenges to implementation, including those unique to the VCS context.

### 3.1 MECC implementation and VCS delivery with seldom‐heard groups

The VCS delivery model provided a unique point of departure from examples of MECC implementation identified nationally. The data highlighted a strong level of fit between MECC principles and organisational ethos for the majority of VCS delivery partners involved in the research, leading to high levels of reported motivation to take part:

‘We help change people’s lives. We’re a little building block and MECC totally fits in with that.’ (R39, VCS Lead—interview)

One key feature of VCS delivery was wide observed diversity and extensive programme reach in terms of partners involved, approach taken, activities delivered and communities engaged. Building on existing local relationships, this formed an important part of the rationale behind encouraging VCS‐led implementation by the local government programme team:

‘I know those communities and the reach [VCS partners] have. If you sit within public health or local authority, quite often you’re removed from those types of communities. So to me it was a perfect way to … thread that together.’ (R3, MECC Delivery Lead—interview)

The analysis highlighted a wide range of client groups accessed through the VCS delivery model. These included people with learning disabilities and difficulties, refugees and asylum seekers, the LGBTQ + community, young carers, older people and those experiencing dementia, young mothers and women experiencing mental health problems. The potential for networking and collaboration across this landscape was an important part of MECC’s perceived value to VCS partners:

‘We wanted to be part of a consortium delivering the same ideology.’ (R14, VCS Lead—workshop)

Despite high levels of motivation to take part, VCS delivery partners reported mixed views related to early expectations and experience of MECC. The findings illustrated a range of administrative and planning complexities that were difficult to navigate locally, including outcomes reporting requirements and training booking systems. Collaborative aspects were reported to have been less central to delivery than anticipated, leading to early recommendations for the introduction of additional, formal networking elements:

‘With the name of the programme being Make Every Contact Count, where is the networking?’ (R8, Client—focus group)

### 3.2 Positive organisational outcomes

The findings articulated a range of positive outcomes for MECC partner organisations. MECC was commonly referred to as having reinforced or validated existing practice, providing a framework to underpin work that organisations were already doing or enabling them to go further in their approach to supporting behaviour change for clients:

‘It provides the authenticity to do something we’ve always done but as part of something bigger … it’s no longer just me being nosey.’ (R38, internal council service—interview)

Examples were provided of positive impact on organisational processes, including the integration of MECC principles into initial client assessments and staff training, as well as on organisational culture through the encouragement of an atmosphere where staff could speak more freely about their health and wellbeing:

‘Because we’ve talked about mental health in the workplace and things like that, lots of conversations … about carers looking after their own health… I think it’s helped embed an atmosphere where people are freer to talk about those things. And … not just virtue sharing but saying, ‘Well, I find it really hard to do that’…’ (R22, VCS Lead—interview)

One key area of perceived benefit to partner organisations was the access to training and subsequent enhanced practice for frontline workers and volunteers. Frontline workers reported increased knowledge on key MECC topics, alongside feeling more confident in raising these topics with clients. The most commonly reported example of practice change was workers being more ‘proactive’, with MECC providing justification to go ‘one step further’ during everyday contacts. Some participants self‐reported an increased frequency of healthy lifestyle conversations with those around them. Others reported more ‘meaningful’ interactions, improved conversation and facilitation skills, or the provision of more ‘appropriate’ responses to clients in crisis:

‘MECC helped me as an individual respond appropriately to several people experiencing suicidal thoughts.’ (R1, VCS Lead—workshop)

‘The whole thing of being a self‐advocacy group is speaking up for people … what it’s helped me to do is, I think I’ve got a broader look … at what gives people good lives … MECC helped us look at things in a different way.’ (R4, VCS Lead—focus group)

Visible in the second quote, MECC was also linked to a perceived re‐framing towards a broader perspective on what gives people ‘good lives’ (R4).

### 3.3 Client outcomes

#### 3.3.1 Knowledge, awareness and behaviour change

Described as a ‘major learning curve’, client focus group participants described enhanced knowledge and awareness on a range of issues related to everyday health behaviours. Food and nutrition were observed as a common area of new knowledge, alongside physical activity (including age‐appropriate exercise), alcohol and sleep routines.

Reported outcomes related to food and nutrition included eating healthier meals, reduced consumption of energy drinks and fizzy drinks, expanded diets, new experiences related to cooking and food handling, and an increase in social cooking and eating activities:

‘It was very informative, we all felt quite elated really … I do now check labels in the shops, especially fat and sugar contents. Previously I would disregard the labels, I wouldn’t even look at them.’ (R42, women’s wellbeing group)

‘I think the main thing for me has been expanding my diet … I started off with a really restricted diet, I only touched the likes of potatoes, cottage pie, just a small group … but then eventually I’ve expanded into pastas … omelettes have become a regular part of my diet … that’s the big thing for me, that’s the difference that both myself and my family have seen.’ (R27, young people’s group)

‘We started doing the cooking on a budget thing, which eventually became Come Dine with Us … cooked our own stuff and now everyone’s … much more aware and self‐conscious of what to watch out for in these different products and how to cook a healthy meal.’ (R26, young people’s group)

Related to physical activity, participants in all three focus groups reported taking part in more physical and outdoor activities, and all three were in the process of setting up their own walking groups as a follow‐on outcome of MECC. Some participants had already seen the benefits of increased physical activity:

‘I’ve seen a lot of difference because I used to have back problems and I’ve lost a little bit of weight as well.’ (R45, women’s wellbeing group)

‘Obviously being outdoors has so many different health benefits, it’s unbelievable … Walking is good for your mental health, it’s good to clear your mind … It feels great.’ (R8, young people’s group)

#### 3.3.2 Wider social and community inclusion

In addition to health and wellbeing behaviour change, focus group participants reported wider outcomes related to social networks, community inclusion and leadership skills. Some were directly linked to MECC, such as peer delivery of MECC sessions, while others came about through a combination of MECC and existing VCS partner activity. Reported outcomes included making friends, increased confidence and a greater awareness of provision in the local community:

‘I think it’s been good because I’ve got to know people that I hadn’t known before … through the MECC I’ve made more friends … cos they come out walking with us as well … I think it’s got more people like us out and about.’ (R32, learning disabilities group)

‘Another thing that’s coming up is we’re planning on a walking project … which I’ve been given the big part of the lead … I’ve already done the planning stages … all the training … basically the health and safety side of walking … your risk assessment, the right things to take outside, to be prepared for all sorts of weather … We’re all prepared, I’ve planned the routes.’ (R26, young people’s group)

#### 3.3.3 Adaptation of MECC for seldom‐heard groups

The findings highlighted the significant time and capacity spent by VCS partners and local authority leads in adapting existing MECC resources to the needs of specific client groups. Examples included creating resources in additional languages or visual rather than written form, and working with clients to design practical or creative activities on key MECC themes. The observed close relationships and regular contact between many delivery partners and their clients enabled the development of creative approaches such as drama, group sports, communal cooking and eating, and even peer intervention models. This led to diverse, varied and tailored application of MECC across VCS delivery partners; with new areas of knowledge and behaviour change closely linked to priority interest areas for each specific client group.

### 3.4 Challenges and barriers to VCS implementation

Balanced against the identified benefits of VCS delivery, the data provided a rich picture of barriers and challenges to successful MECC implementation at different levels including national programme, local programme, VCS sector, partner, frontline worker and client. These are summarised in Table 5.

**TABLE 5:** Summary of identified barriers and challenges to MECC implementation

National programme‐level	Inclusivity and accessibility: A westernised/generalised model? Lack of attention to individual circumstances (e.g. culture, class, income, relationships) Long‐term sustainability Measuring national impact
Local programme‐level	Time‐limited funding Planning, administration and resources VCS delivery model details (e.g. monitoring and reporting requirements, funding parameters and restrictions) Strategic leadership and organisational commitment (internal and external) Long‐term support, transfer of knowledge and links to wider networks Evaluation and monitoring outcomes Support for collaboration and partnership working
VCS sector‐level	Funding and organisational uncertainty Weak governance and lack of infrastructure funding Fluctuating workforce (including reliance on volunteers) Lack of funded opportunities for partnership working
Partner‐level	Staff capacity and workload pressures Part‐time working and reliance on casual staff/volunteers Existing reporting and recording systems Lack of financial flexibility to absorb additional/unexpected costs Nature of client contact (e.g. one‐off vs. regular)
Frontline worker‐level	Motivation to take part (including perceptions of value and relevance to role) Background and existing knowledge Own health and wellbeing Time and workload pressures Language and cultural barriers
Client‐level	Complexity of existing health and wellbeing issues Individual interest and attitudes towards change Background and existing knowledge Wider individual circumstances (incl. financial situation, language and cultural factors) Wider factors (incl. benefits assessments)

Key challenges at national programme‐level included the accessibility and suitability of MECC training and resources for specific seldom‐heard groups, such as those with English as a secondary language or those with learning difficulties. Flexibility was a crucial factor in successful implementation, as it enabled the development of bespoke, tailored programmes in response to locally identified need, interest and constraints.

Local programme‐level challenges were also illustrated, including planning requirements and financial resources allocated to MECC. VCS sector and local partner‐level barriers included variations in organisational commitment, capacity and workload pressures, staff turnover and the fluctuating VCS volunteer base. Many such challenges were linked to the wider context of sector‐level financial uncertainty, which in turn led to concerns around MECC legacy, long‐term sustainability and transfer of knowledge across the VCS workforce. Substantial barriers were also reported in relation to measuring MECC outcomes, with some partners feeling unable to provide sufficient capacity to fulfil MECC reporting requirements.

At frontline worker‐level, while perceptions of the overall value of MECC were generally high for those involved in the evaluation, there were some notable individual differences. Where participants did not identify positive outcomes, a range of explanatory factors were identified. These included perceived relevance, background and existing knowledge, role and service focus (including nature and frequency of client contact), workload/capacity, and perceptions of client relationships—including the potential for MECC to negatively impact on these.

The data also illustrated several client‐level challenges. These included individual differences in motivation, interest and attitude towards change, alongside complexity of existing health and wellbeing issues, wider personal circumstances (including financial situation), language barriers and cultural differences. Some of these issues raised a perception of MECC ‐ in its original form ‐ as a privileged or westernised approach:

‘The only word I would probably be able to use to describe it … is judgemental in that it’s expecting the same level of engagement from marginalised parts of society that you would expect from people who have grown up with privilege and I felt really mixed about that.’ (R21, VCS frontline worker—interview, describing initial thoughts on MECC)

## 4 DISCUSSION

### 4.1 Advantages and disadvantages of a VCS delivery model of MECC

The focus on the VCS as central delivery partner of a national health behaviour change programme provided a unique context within which to explore implementation and early impact from the perspective of partner organisations, frontline workers, volunteers and clients.

The findings highlighted clear successes of VCS implementation, including the diversity and extensive reach of the programme which engaged a range of seldom‐heard groups. Close, trusted relationships and opportunities for regular client contact held by many partners created opportunities to develop creative, co‐produced and relatively long‐term interventions. Based on the current findings, we suggest that VCS‐led implementation of brief interventions may hold the potential to reach a broader range of clients, and in more diverse ways, than more traditional forms of implementation by clinical practitioners. This supports existing literature which highlights the ability of VCS organisations to provide flexible, trusted services leading to improved engagement with those who are marginalised (Flanagan & Hancock, 2010; Goopy & Kassan, 2019; Powell et al., 2017).

There were however notable trade‐offs associated with VCS delivery of MECC brief interventions. Conceptualised as a ‘double‐edged sword’, challenges included the need for substantial adaptation of resources and additional work—from both the MECC programme team and VCS partners—to improve accessibility to specific client groups. Additional administrative implications included the need for flexibility to respond to a fluctuating workforce and volunteer base, with a high prevalence of part‐time working alongside limited organisational capacity to release workers for training. The findings provide useful learning related to both the benefits and support requirements of increased VCS involvement in delivery of brief interventions, as well as other public sector initiatives such as social prescribing.

In the current study, much of MECC’s reported success was attributed to the knowledge, enthusiasm and hard work of individual partners and delivery team members. While specific aspects of MECC were highly regarded—such as its flexibility and simplicity of the messages included in training sessions—the data also painted a rich picture of barriers and challenges to successful implementation. Many of the benefits and challenges identified here support findings reported in the wider literature (Bull & Dale, 2020; Nelson et al., 2012; Patten & Crutchfield, 2016). As highlighted in Table 5 however, additional layers of complexity were associated with the VCS delivery model, including sector‐level funding uncertainty, high staff turnover and reliance on volunteers, and the specific needs and requirements of such a diverse client base. These required local programme leads to help address and navigate barriers faced by individual partners and the VCS sector more widely.

### 4.2 Legacy and long‐term sustainability

The evaluated MECC programme can be understood as a relatively short‐term approach to behaviour change, having received time‐limited funding over 2 years. Longer‐term ambitions of continued, unfunded roll‐out by its delivery partners raise questions regarding long‐term sustainability and learning transfer. The need for high‐level, strategic commitment to ensure MECC’s long‐term success emerged as a key theme in this study, mirroring findings elsewhere in the literature (Dewhirst & Speller, 2015; Nelson et al., 2012).

The challenges of sustaining behaviour change for both practitioners and clients beyond an intervention's initial timeframe are well‐documented (Dombrowski et al., 2016; Kwasnicka et al., 2016). In the current study, the importance of long‐term sustainability was visible from both the client and frontline worker perspective, in relation to maintaining individual behaviour change as well as the need to regularly update and reinforce key public health messages. The significant role of wider support networks was also highlighted, anchored in a conversation about cross‐sector reduction in capacity to support health‐related behaviour change in recent years. This raises important questions around who will be responsible for driving MECC forward in the longer term and the wider capacity required to support it locally.

### 4.3 MECC as a universal public health solution

The findings reported here encourage wider discussion around MECC as a conceptual model, including its efficacy and inclusivity as a national model of health behaviour change. The research demonstrated that MECC in its original format may not be accessible for seldom‐heard client groups or those with complex needs, with substantial tailoring required by those delivering interventions to enable meaningful participation. Whether this is a realistic expectation for those driving the initiative locally, or should instead be considered a crucial part of MECC’s national remit, is open to debate. In addition, limited consideration of the impact of wider contextual factors such as income, family situation, stress and culture on health behaviour led to a perception of MECC as a westernised or ‘privileged’ approach to behaviour change. If MECC or other behaviour change interventions are intended to engage fully with seldom‐heard groups, this work offers insight into areas for future development and investment. This is essential to ensuring that support for health behaviour change is accessible and embedded in the lives of those such interventions are seeking to improve.

### 4.4 Understanding and measuring brief intervention outcomes

Finally, while the research documented positive early outcomes of MECC, it also raised a series of issues related to programme implementation, monitoring and evaluation. The findings emphasise the need for simple, appropriate and realistic reporting requirements. There was wide variation in motivation and commitment to monitoring outcomes among VCS partners, alongside practical issues related to the robustness of recording systems and their accessibility to external evaluation. Issues related to the visibility of MECC as a standalone programme with separate, attributable and measurable outcomes—as with many brief interventions and preventative approaches—presented further, substantial barriers:

‘I think people don’t really see it as a programme. They just see it as a nice walk on a Friday afternoon … and that’s the whole idea of it of course.’ (R30, VCS frontline worker)

### 4.5 Study limitations

While this research provides some promising early findings, it shares several limitations with earlier work on brief interventions and the role of VCS organisations in service delivery. Limitations include the focus on one specific programme in one geographical location at one time‐point, which may or may not prove transferable to other contexts. In addition, a lack of accessible reporting systems or research team involvement in the programme's planning stages created an unavoidable reliance on qualitative methods and process evaluation. These points reflect challenges reported elsewhere and re‐emphasise the call for long‐term, strategic evaluation of MECC alongside investment in shared, accessible evaluation tools (Nelson et al., 2012; Patten & Crutchfield, 2016).

Regarding the focus on seldom‐heard groups, it was beyond the scope of the evaluation to examine the extent to which VCS partners meaningfully engaged their target client base. Even with the heightened focus on under‐represented groups within the studied MECC delivery model, we can assume that there will have been a significant cohort of people who fell outside the programme's reach. Focus group participant status as a member of a seldom‐heard group was inferred from their membership of a particular VCS organisation, rather than being objectively measured. This decision was made based on ethical considerations and in light of the wider challenges of engaging ‘hard‐to‐reach’ groups in research (Rockcliffe et al., 2018; Waheed et al., 2015). Similarities shared by the VCS organisations who did not opt‐in to the evaluation raise future research questions around the feasibility of delivering such interventions with client groups who may be facing an immediate crisis, or a complex or progressive diagnosis—for example those experiencing homelessness, eating disorders or dementia.

### 4.6 Conclusion and Recommendations

This paper contributes to learning related to MECC and other brief intervention programmes, through the detailed analysis of a VCS‐led delivery model in practice. While this has been demonstrated to add value through diversity, reach and the close nature of client contact, it has simultaneously been illustrated to create a uniquely challenging environment for implementation. This highlights a need for system‐level analysis and comparison of different brief intervention delivery models, to expand our understanding of barriers, facilitators and programme reach beyond traditional implementation contexts.

Building on the multi‐layered identification of barriers and challenges to implementation, the findings led to several recommendations for local and national implementation of MECC and other brief intervention programmes. Recommendations include the development of diverse and accessible resources at a national level that are suitable for different backgrounds, cultures and levels of need—alongside increased opportunities for sharing learning among delivery partners. Furthermore, there is a need to develop robust, shared evaluation and monitoring tools which take into account wider pressures on delivery partners and are simple to use, realistic and accompanied by supporting guidance or training. Finally, this work challenges a critical assumption of MECC—and many other behaviour change programmes—that contact with those in need of support should be facilitated through standardised public health messages rather than tailored, individualised support co‐produced with those whose lives it seeks to change.

## CONFLICT OF INTEREST

The authors declare that there is no conflict of interests. The research was undertaken externally by the academic research team, independent from the funding organisation.

## AUTHOR CONTRIBUTION

All authors contributed to the conceptualisation and design of the study. Mapping workshop data was collected by DH, RW, KB and GC. Interviews were undertaken by DH, KB and GC. Focus group discussions were facilitated by DH, KB, HH and GC. DH and RW coded and analysed the data. All authors contributed to interpreting the data. The first draft of the manuscript was written by DH. All authors contributed to the development, reviewing and editing of the paper. All authors read and approved the final manuscript.

## Data Availability

The data that support the findings of this study are available from the corresponding author upon reasonable request. The data are not publicly available due to privacy or ethical restrictions.
